# ASO Author Reflections: Multimodality Treatment in Esophageal Signet Ring Cell Adenocarcinoma

**DOI:** 10.1245/s10434-019-07924-5

**Published:** 2019-10-21

**Authors:** Sander J. M. van Hootegem, Bas. P. L. Wijnhoven

**Affiliations:** Department of Surgery, Erasmus MC, University Medical Center, Rotterdam, The Netherlands

## Past

The aggressive and infiltrating behaviour of signet ring cell (SRC) tumours may be associated with poor prognosis.^1^ Although multimodality treatment has become the standard of care for oesophageal and junctional adenocarcinoma, the role of neoadjuvant therapy has been under debate for SRC tumours. Still little is known about the exact impact of SRC histology on the efficacy of multimodality treatment in oesophageal and junctional adenocarcinoma.^1^,^2^ We, therefore, assessed the impact of SRC histology on response to neoadjuvant therapy and survival.

## Present

Despite SRC histology not being an independent predictor for overall or disease-free survival in this study, tumours with an SRC component had a higher rate of irradical (R1/R2) resections and more advanced pathological T-stage after neoadjuvant therapy compared with non-SRC tumours.^3^ SRC tumours had a higher rate of irradical resections and a more advanced pathological T-stage, resulting in worse locoregional recurrence-free survival after receiving nCT. On the contrary, following nCRT, no statistically significant differences were found between groups for overall and disease-free survival and pathological characteristics. Moreover, the SRC group had worse disease-free survival compared with the non-SRC group after receiving nCT. These results indicate that nCRT could achieve comparable locoregional control, recurrence rates, and survival as in patients with non-SRC tumours. Given the behavioural features of SRCs, locoregional control could be of major importance.

## Future

As biopsies are not always representative for the pheno- and genotype of the entire tumour, they may not be accurate enough to determine the percentage of SRC present.^1^ However, Bekkar et al. compared nCRT with surgery alone in > 50% SRC tumours and found a significant survival benefit and lower rates of recurrence after nCRT.^4^ This study underlines the findings of the present study that favourable oncologic outcomes after nCRT could be achieved in SRC tumours, independent of percentage.

Possible intensification of locoregional therapy, however, may still be needed, and larger, prospective studies should be conducted stratifying for SRC histology to establish nCRT as optimal treatment regimen for oesophageal SRC tumours.
